# Shikonin selectively induces apoptosis in human prostate cancer cells through the endoplasmic reticulum stress and mitochondrial apoptotic pathway

**DOI:** 10.1186/s12929-015-0127-1

**Published:** 2015-04-01

**Authors:** Rishi Kumar Gara, Vikas Kumar Srivastava, Shivali Duggal, Jaspreet Kaur Bagga, MLB Bhatt, Sabyasachi Sanyal, Durga Prasad Mishra

**Affiliations:** Cell Death Research Laboratory, Endocrinology Division CSIR-Central Drug Research Institute, Lucknow, 226031 India; Center for Cancer Research, UTHSC, Memphis, TN USA; Department of Radiotherapy, King George Medical University, Lucknow, 226003 India; Division of Biochemistry, CSIR-Central Drug Research Institute, Lucknow, 226031 India

**Keywords:** Shikonin, Prostate cancer, Endoplasmic reticulum stress, Calpain pathway, ROS, Mitochondrial dysfunction

## Abstract

**Background:**

Despite the recent progress in screening and therapy, a majority of prostate cancer cases eventually attain hormone refractory and chemo-resistant attributes. Conventional chemotherapeutic strategies are effective at very high doses for only palliative management of these prostate cancers. Therefore chemo-sensitization of prostate cancer cells could be a promising strategy for increasing efficacy of the conventional chemotherapeutic agents in prostate cancer patients. Recent studies have indicated that the chemo-preventive natural agents restore the pro-apoptotic protein expression and induce endoplasmic reticulum stress (ER stress) leading to the inhibition of cellular proliferation and activation of the mitochondrial apoptosis in prostate cancer cells. Therefore reprogramming ER stress-mitochondrial dependent apoptosis could be a potential approach for management of hormone refractory chemoresistant prostate cancers. We aimed to study the effects of the natural naphthoquinone Shikonin in human prostate cancer cells.

**Results:**

The results indicated that Shikonin induces apoptosis in prostate cancer cells through the dual induction of the endoplasmic reticulum stress and mitochondrial dysfunction. Shikonin induced ROS generation and activated ER stress and calpain activity. Moreover, addition of antioxidants attenuated these effects. Shikonin also induced the mitochondrial apoptotic pathway mediated through the enhanced expression of the pro-apoptotic Bax and inhibition of Bcl-2, disruption of the mitochondrial membrane potential (MMP) followed by the activation of caspase-9, caspase-3, and PARP cleavage.

**Conclusion:**

The results suggest that shikonin could be useful in the therapeutic management of hormone refractory prostate cancers due to its modulation of the pro-apoptotic ER stress and mitochondrial apoptotic pathways.

**Electronic supplementary material:**

The online version of this article (doi:10.1186/s12929-015-0127-1) contains supplementary material, which is available to authorized users.

## Background

Prostate cancer is one of the leading causes of urino-genital cancer related deaths in men [1]. Despite the initial response to androgen deprivation, the disease gradually progresses to a hormone-refractory state due to cumulative genetic alterations resulting in progressive clinical deterioration [2]. Despite the recent advances in diagnostic methods and improvement in treatment strategies mostly using androgen pathway inhibitors, the prognosis of hormone refractory prostate cancers in advanced stages remains largely unsatisfactory [3,4]. Therefore there is a urgent need for expedited development of effective therapeutic agents against hormone refractory prostate cancers.

Aberrant accumulation of unfolded/misfolded proteins and lipids or sudden changes of the endoplasmic reticulum Ca^2+^ homeostasis leads to a cellular adaptive response known as endoplasmic reticulum stress (ER stress). However, excessive ER stress is believed to be intricately associated with oxidative stress and mitochondrial dysfunction, resulting in apoptotic cell death [5-7]. Therefore the therapeutic modulation of the pro-apoptotic ER stress could be a potential strategy for chemo-sensitization of hormone refractory prostate cancer cells [8-10].

Shikonin, a natural naphthaquinone compound from the herb *Lithospermum erythrorhizon* is known to act on a variety of molecular targets associated with carcinogenesis and shows similar potency towards drug sensitive and drug-resistant cancer cell lines [11-17]. Furthermore, Shikonin is used as a food additive in many countries and has favorable toxicity, pharmacokinetic and pharmacodynamic profiles [15,16,18]. However its effects on pro-apoptotic-ER stress in hormone refractory prostate cancer cells is unknown. Therefore in the present study, we examined the effects of Shikonin on DU-145 and PC-3 prostate cancer cells and investigated the molecular mechanisms involved in the process.

## Methods

### Materials and reagents

Hormone refractory prostate cancer cell lines DU-145, PC-3 and PrEC, a normal prostate cell type were purchase from ATCC (ATCC; Manassas, VA, USA) and Lonza (Walkersville, MD USA) respectively. The details of the cell lines used in this study are summarized in the (Additional file 1: Table S1). RPMI-1640 media and fetal bovine serum (FBS) were purchased from Gibco Life Technologies (Life Technologies, Inc., Rockville, MD, U.S.A.). Shikonin and Salubrinal (ER stress inhibitor) were purchased from Calbiochem (San Diego, CA, U.S.A.). 4′,6-diamidino-2-phenylindole (DAPI), and 5,5′,6,6′-tetrachloro-1,1′,3,3′-tetraethyl- benzimidazolylcarbocyanine iodide (JC-1) were obtained from Invitrogen (Carlsbad, CA, U.S.A.). Trypsin, streptomycin, penicillin, N-acetyl cysteine (NAC), glutathione (GSH) and Catalase were obtained from Sigma Chemical Co. The antibodies used in this study were purchased from Santa Cruz Biotechnology Inc. (Santa Cruz, CA, U.S.A.). Caspase colorimetric assay kits were purchased from Millipore (Billerica, CA, USA). Rest of the chemicals used in the study were from Sigma (St. Louis, MO, U.S.A.) unless otherwise stated.

#### Cell culture and treatment

DU-145, PC-3 and PrEC cells were grown in RPMI 1640 medium (Life Technologies, Inc., Rockville, MD) with 10% heat-inactivated fetal bovine serum (FBS; Life Technologies, Inc.) or DMEM (Life Technologies, Inc.) supplemented with 10% fetal bovine serum (FBS) (Hyclone, Logan, UT, USA) at 37°C with 5% CO2 incubator. Stock of Shikonin was prepared in DMSO and stored in −20°C, cells were treated with different concentration and time periods with Shikonin for different experiments.

#### Cell viability assay

Cell viability was measured using the CCK-8 assay kit in (PC-3 and DU-145) hormone refractory prostate cancer cells and PrEC cells as per the manufacturer’s instructions. Cells were treated with Shikonin for various time points, at the end of treatment, the absorbance was read using a Fluostar Omega Spectrofluorimeter (BMG Technologies, Offenburg, Germany). All the experiments were repeated at least thrice.

#### Cell proliferation assay

Cellular proliferation was measured by measurement of bromodeoxyuridine (BrdU) incorporation into DNA using a nonradioactive colorimetric assay using ELISA (Roche Applied Science, Indianapolis, IN) as per the manufacturer’s instructions. All the experiments were repeated at least thrice.

#### Flowcytometry

Assessment of DNA fragmentation was done using the TUNEL assay according to a previously standardized procedure [19]. Briefly, cells were harvested and fixed in freshly prepared 4% para-formaldehyde in PBS for 30 min at 4°C and then in 70% ethanol for 1 h at 4°C. Subsequently the fixed cells were permeabilized using 0.2% Triton X-100 in 0.1% sodium citrate. The DNA labeling mixture containing terminal deoxynucleotidyl transferase was then added. Cells were incubated overnight at room temperature and washed twice with PBS. Controls were resuspended in the TUNEL reaction mixture containing fluorescent dUTP without terminal deoxynucleotidyl transferase. Finally the analysis was carried out in a BD LSR flow cytometer (Becton–Dickinson, San José, CA).

#### Measurement of reactive oxygen species

For measurement of reactive oxygen species, the cell permeant probe CM-H2DCFDA was used. The dye was dissolved in dimethyl sulfoxide, and dilutions were made in culture medium. Cells were seeded overnight in 6-well plates with various treatments. At the end of treatments the cells were incubated with 20 μM of the fluorescent probe 2′,7′-dichlorofluorescein diacetate (DCF-DA) for 30 min. At the end of the incubation period adherent cells were trypsinized and collected. After washing twice with phosphate-buffered saline (PBS, pH 7.4) the fluorescence was monitored at an excitation wavelength of 488 nm and an emission wavelength of 530 nm in a Fluostar Omega Spectrofluorimeter (BMG Technologies, Offenburg, Germany) over a period of time. For each experiment, fluorometric measurements were performed in triplicate and expressed as fluorescence intensity units.

#### Measurement of intracellular Ca^2+^concentration

The intracellular Ca^2+^ levels in DU-145 and PC-3 cells were determined using flourimetry with the Fluo-4 AM dye (Invitrogen Carlsbad, CA, U.S.A.). Cells were cultured in specialized 96-well plates and loaded with 5 μM of Fluo-4 AM fluorescent dye for 30 min at 25°C. Experiments were performed in Hanks’ Balanced Salt Solution (HBSS) solution containing (mM); NaCl, 142; KCl, 5.6; MgCl_2_, 1; CaCl_2_, 2; Na_2_HPO_4_, 0.34; KH_2_PO_4_,0.44; HEPES, 10; glucose, 5.6; buffered to pH 7.4 with NaOH. The Ca^2+^ −free HBSS had the same constituents as HBSS solution, but with no CaCl_2_ and with EGTA 1 mM added to eliminate any possible calcium contamination. Fluorescence measurements were performed at an excitation of 488 nm and an emission of 522 nm Fluostar Omega Spectrofluorimeter (BMG Technologies, Offenburg, Germany). All the experiments were repeated at least thrice.

#### Calpain activity assays

DU-145 and PC-3 cells were cultured on 24-well plates and pretreated with BAPTA, a Ca^2+^ chelator or calpeptin and an inhibitor of calpain for 1 h. Then, cells were loaded with 40 M Suc- Leu-Leu-Val-Tyr-AMC calpain protease substrate (Biomol, USA,) and treated with shikonin to the indicated time at 37°C in a humidified 5% CO2 incubator. Proteolysis of the fluorescent probe was monitored by a Fluostar Omega Spectrofluorimeter (BMG Technologies, Offenburg, Germany) with filter settings of 360 nm for excitation and 460 nm for emission. All the experiments were repeated at least thrice.

#### Western blotting analysis

The protein content of the control and treated cell extracts was measured by the Bradford assay. The western blotting was done as per a previously standardized protocol [19]. Briefly, each of the samples 50 μg of protein were electrophoresed on 10–12% SDS–PAGE gels and transferred to nitrocellulose membranes. Membranes were blocked, incubated with primary antibodies at the appropriate concentration, and subsequently incubated with horseradish peroxidase-conjugated goat anti-rabbit IgG or goat anti-mouse IgG (1:5000 dilution). Labeled bands were detected by Immobilion western chemiluminescence horseradish peroxidase kit and images were captured. Densitometric analysis for determination of relative protein expression was done using a Doc image system (Bio-Rad, Laboratory, UK) with β-actin as loading control.

#### Measurement of mitochondrial membrane potential

The integrity of mitochondrial membrane potential (Δψ) was measured by JC-1 (5, 5′, 6, 6′-tetrachloro-1, 1′, 3, 3′-tetraethyl-benzimidazolylcarbocyanine iodide; T-3168; Molecular Probes, Eugene, OR), a cationic dye that exhibits potential-dependent accumulation in mitochondria, indicated by a fluorescence emission shift from green (527 nm) to red (590 nm). With normal mitochondrial function, Δψ is high and red fluorescence is predominant. After injury to mitochondria, Δψ is reduced and an increase in green fluorescence is observed. At the end of the treatments, cells were washed and incubated with JC-1 dye at the concentration of 10 μg/ml in media for 15 min at 37°C. The presence of depolarized mitochondria was identified by fluorimetry. The ratio of the reading at 590 nm to the reading at 530 nm (590:530 ratio) was considered as the relative (Δψ) value. All the experiments were repeated thrice.

#### Caspase activity assay

To measure caspase-9 and caspase-3 activities, cell lysates were prepared from cells treated with Shikonin for 24 h or with various treatments and analyzed using the caspase-9 and caspase- 3 colorimetric activity assay kits as per the manufacturer’s instructions. All the experiments were repeated thrice.

#### Statistical analysis

The data shown are a summary of the results from at least three independent experiments and are presented as the means ± standard error (SEM.). A statistical evaluation of the results was performed with one-way analysis of variance (ANOVA). The results were considered significant at a value of p < 0.05.

## Results

### Shikonin inhibits proliferation of prostate cancer cells without affecting normal prostate epithelial cells

**Figure 1:**
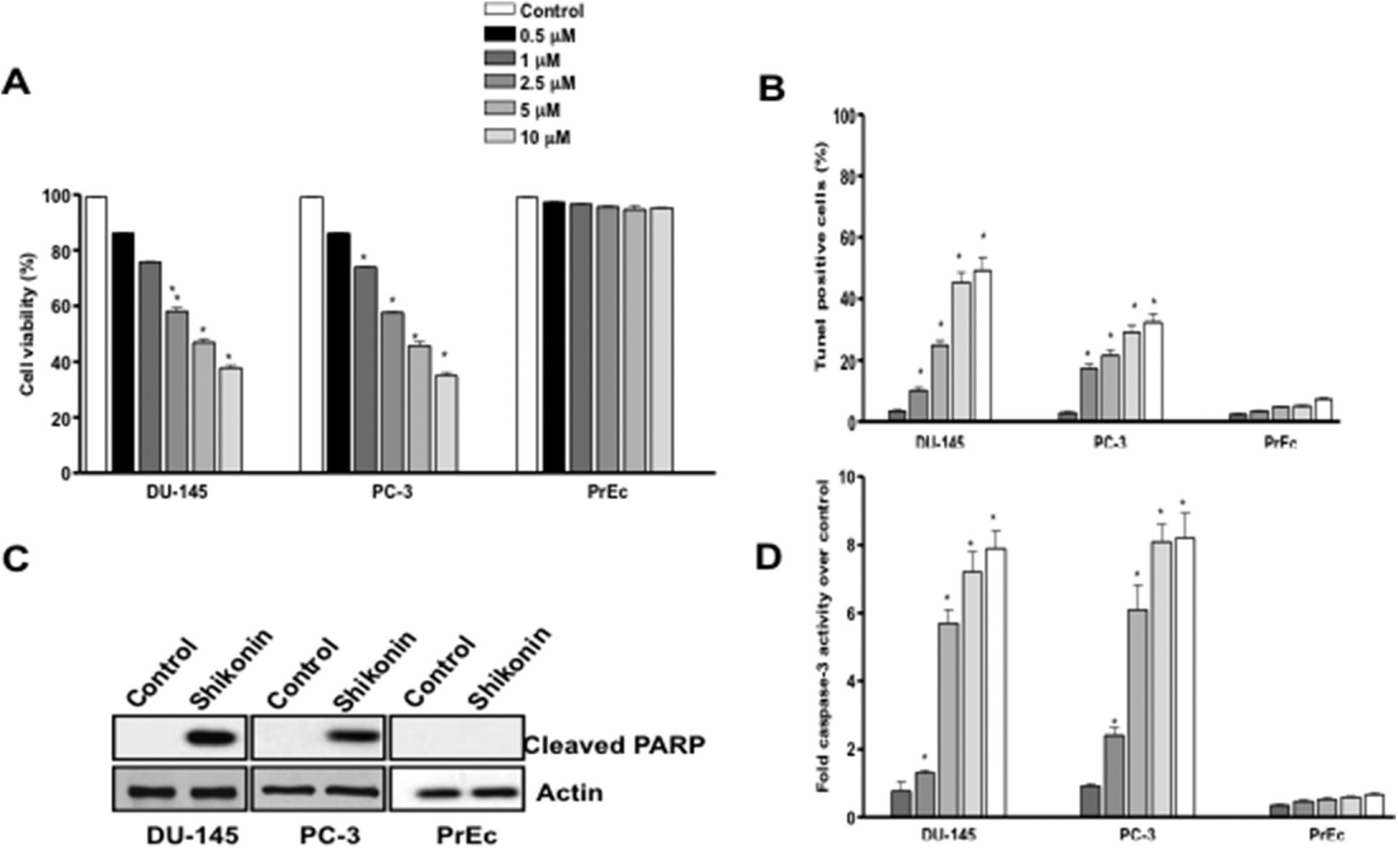
**Shikonin treatment selectively induces apoptosis in prostate cancer cells. (A)** Shikonin treatment inhibits prostate cancer cell viability without affecting normal prostate epithelial cells. DU-145, PC-3 and PrEC cells were treated with various doses (0.5, 1, 2.5, 5 and 10 μM) of Shikonin for 24 h and CCK-8 assay was used for the measurement of cell viability. **(B)** Shikonin treatment induces DNA fragmentation in prostate cancer cells. Cells were treated with various doses (0.5,1,2.5, 5 and 10 μM) of Shikonin for 24 h and TUNEL assay was done using flow cytometry and the % TUNEL positive cells were measured. **(C)** Shikonin treatment induces PARP activation. Cells were treated with 2.5 μM of Shikonin for 24 h and the cell lysates were analyzed by western blotting for the detection of cleaved PARP, a marker for PARP activation.** (D)** Shikonin treatment induces caspase-3 activation. Cells were treated with various doses of Shikonin (0.5, 1, 2.5, 5 and 10 μM) for 48 h and measurement of caspase-3 activity was done using the CASPASE-3 Colorimetric Activity Assay kit. Data is expressed in means ± SEM and represents the results of three independent experiments (p < 0.05).

Hormone refractory prostate cancer cells (DU-145 and PC-3) were treated with various doses of Shikonin (0.5, 1, 2.5, 5 and 10 μM) for 24 h. Cell viability was determined using the CCK-8 assay kit. The results indicated that the IC50 dose of Shikonin was ~ 5.0 μM in DU-145 cells and ~4.5 μM in PC-3 respectively. Consistent with earlier studies shikonin inhibited viability of a variety of non-prostate cancer cell types while sparing the normal cell types (Additional file 2: Figure S1). Furthermore, Shikonin inhibited proliferation of prostate cancer cells in a dose dependent manner (Additional file 2: Figure S2). While no significant (p > 0.05) effect on cell viability was observed in normal prostate epithelia cells (PrEc). Since a significant (~42%, p < 0.05) decrease in the cell viability was observed in both these cell lines treated with 2.5 μM of dose of Shikonin (Figure 1A), we selected this dose for the mechanistic experiments of the study. DNA fragmentation a characteristic of the apoptotic cells was quantified by the flowcytometry based TUNEL assay in Shikonin treated cells. The results depicted a significant (p < 0.05) increase in the TUNEL positive cells with Shikonin treatment (Figure 1B). Cleavage of PARP, a highly conserved 116 kDa nuclear poly (ADP-ribose) polymerase, is implicated in the apoptotic response of cells. Immunoblotting experiments clearly showed that Shikonin treatment induced PARP activation characterized by the cleaved PARP fragment in DU-145 and PC-3 cells, interestingly we did not observed any PARP clevage in PrEcs (Figure 1C). Activation of caspase-3 like proteases play a crucial role in apoptotic cell death, therefore we next determined the caspase-3 activities in Shikonin treated and DU-145 and PC-3 cells. A ~5.8-fold increase in the caspase-3 activities over control cells was observed in these cell lines (Figure 1D; p < 0.05). Collectively, these results indicated that shikonin inhibited proliferation of the prostate cancer cells through induction of apoptotic cell death.

### Shikonin treatment induces ROS generation, intracellular calcium and ROS-dependent mitochondrial apoptosis in prostate cancer cells

**Figure 2:**
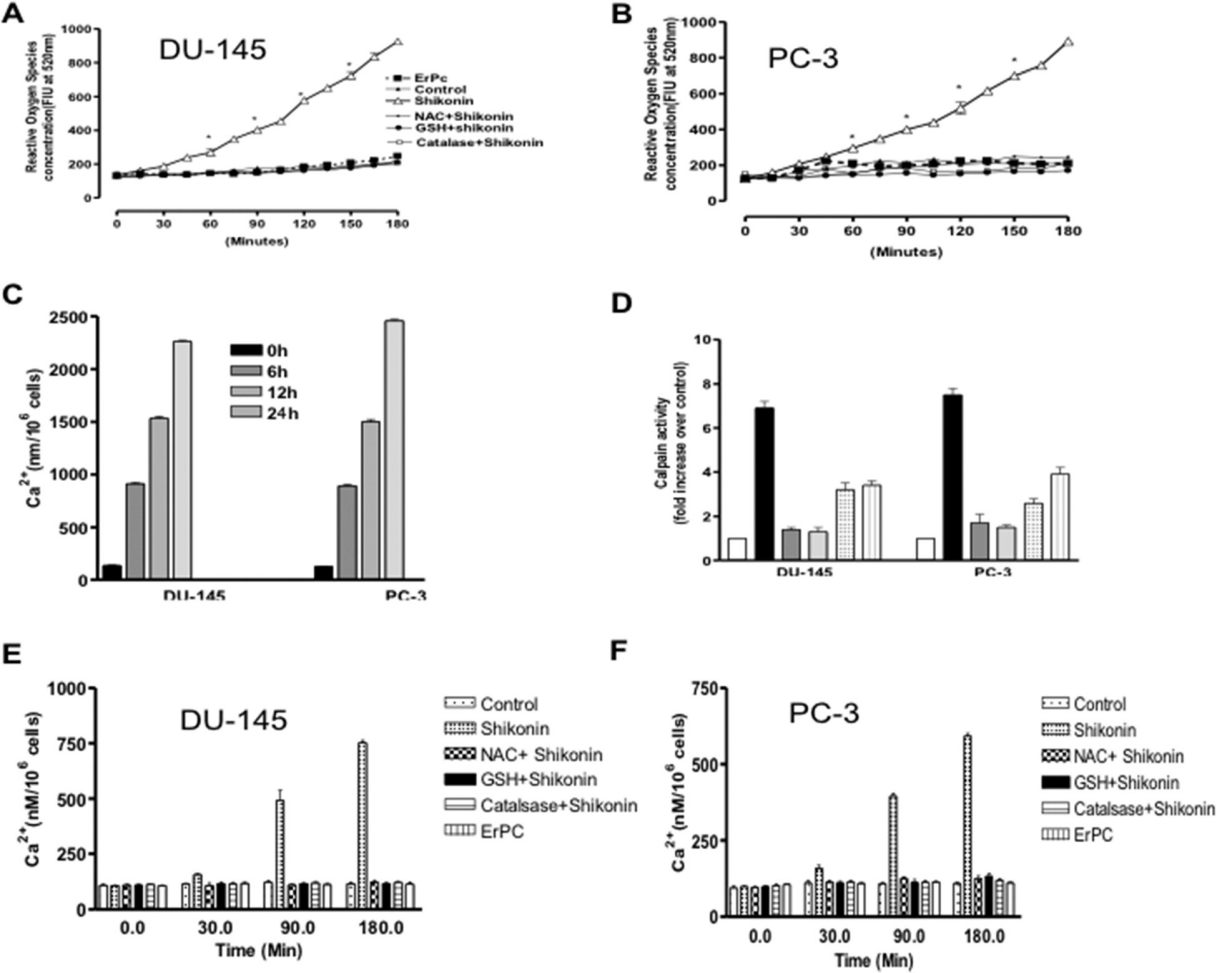
**Shikonin treatment induces ROS generation, increase in Intracellular Calcium in prostate cancer cells. (A-B)** Shikonin induces ROS generation in prostate cancer cells. DU-145** (A)** and PC-3 **(B)** cells were seeded in 6-well plates overnight in the presence or absence of Shikonin, and incubated with DCF-DA 50 μM for 30 min, washed three time with ice cold PBS. Generation of ROS was measured using flourimetry as per standard protocol. **(C)** Shikonin induces increase in intracellular Calcium in Shikonin treated prostate cancer cells. The level of intracellular calcium in DU-145 and PC-3 cells were determined using flourimetry technique in cells using the Fluo-4AM (50 μM/ml) dye in Shikonin treated prostate cancer cells as described in material and methods. **(D)** Shikonin treatment induces calpain activity in prostate cancer cells. Shikonin treated prostate cancer cells were analyzed for calpain activity as described in as described in material and methods. **(E-F)** Antioxidant pretreatment reverses shikonin induced increase in the intracellular calcium. DU-145 and PC-3 cells were pretreated with NAC (20 μM), GSH (10 mM) or Catalase (20 nM) for 2 hours and the ROS inhibitor rescued intracellular calcium release was measured as described in the Methods section. Values are represented as Mean ± SEM, from three independent experiments.

There is a close relationship between ROS and Ca^2+^ in apoptotic signal transduction pathways [20]. Shikonin is known to influence intracellular Ca^2+^ [20] and ROS generation in different cancer cells types [21-23] but similar modulation of intracellular Ca^2+^ in hormonal refractory prostate cancer cells has not been elucidated. Our results indicated that Shikonin treatment induces time dependent ROS generation, which could be rescued with pretreatment of antioxidants (NAC, GSH and Catalase) in both DU-145 (Figure 2A) and PC-3 (Figure 2B) cells. ROS mediated free intracellular Ca^2+^ is known to be involved in ER stress induced apoptotic signaling [24]. Therefore we further investigated for these changes, in DU-145 and PC-3 cell, treated with Shikonin. We observed a marked increase in the intracellular Ca^2+^ levels (Figure 2C) and Calcium-Activated Neutral Protease (Calpain) activity as early as 60 min (Figure 2D) in shikonin treated DU-145 and PC-3 cells. Pretreatment with ROS inhibitors (NAC, GSH and Catalase) attenuated ROS-induced intracellular Ca^2+^ levels in DU-145 (Figure 2E) and PC-3 cells (Figure 2F). Excessive generation of ROS renders the cells oxidatively stressed and impairs membrane proteins, leading to mitochondrial dysfunction and apoptotic cell death [25]. Shikonin treatment induced significant loss of mitochondrial membrane potential (Figure 1A) and modulated pro-anti-apoptotic mitochondrial proteins expression (Figure 1B) in these cells. Our western blot analysis showed that shikonin activated expression of the upstream proteins Bax, cytochrome c as well as downstream caspases 7 and AIF in both prostate cancer cell types (Figure 1B). Moreover, pre-incubation with ROS inhibitors (NAC, GSH and Catalase) attenuated these effect in both cell types (Figure 1C). Collectively these results indicated that Shikonin treatment induces ROS generation, increases in intracellular calcium levels and ROS-dependent mitochondrial apoptosis in prostate cancer cells.

### Shikonin treatment induces oxidative stress mediated induction of ER stress related protein expression

**Figure 3:**
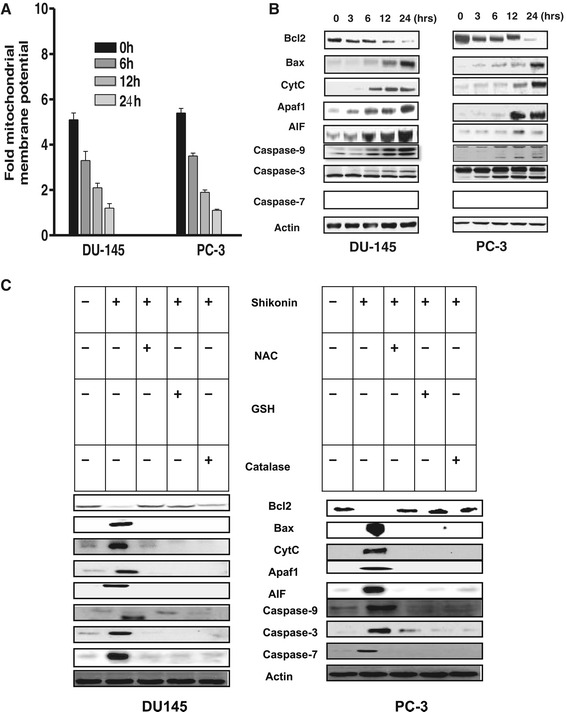
**Shikonin induces ROS dependent mitochondrial apoptosis in prostate cancer cells. (A)** Shikonin modulated mitochondrial membrane potential in DU-145 and PC-3 cells. The integrity of mitochondrial membrane potential (Δψ) was measured by JC-1 mitochondrial dye. Briefly DU-145 and PC-3 cells were treated with 2.5 μM for 0–24 h. Ratio of 590/530 nm was measured by flow cytometry as described in material and methods. Data is expressed in means ± SEM and represent the results of three independent experiments (p < 0.05). **(B)** Shikonin treatment alters expression of mitochondrial proteins in DU-145 and PC-3 cells. Cells were treated with Shikonin for 0-24 h, expression of indicated mitochondrial proteins were performed as described in Methods. Experiments were repeated minimum two-four times. **(C)** Shikonin induced ROS dependent mitochondrial apoptosis in DU-145 and PC-3. Cells were pretreatment with the antioxidants NAC (20 μM) GSH (10 mM) and Catalase (20 nM) and western bolting experiments were performed as described in Methods section. Data is represents the results of three independent experiments.

**Figure 4:**
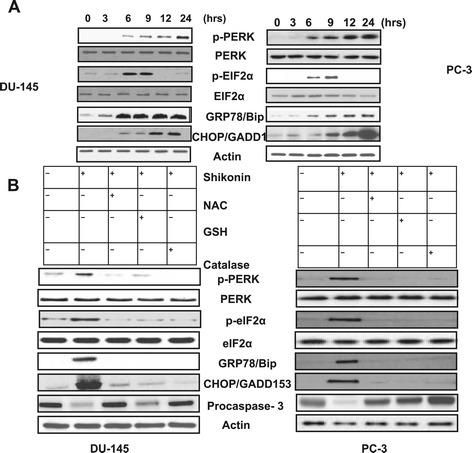
**Shikonin induces reactive oxygen species mediated ER Stress. (A)** Shikonin treatment induces ER stress associated protein expression. Western blot experiments in prostate cancer cells treated with 2.5 μM of Shikonin increased the phosphorylation of PERK, and its substrate eIF2α, increased expression of GRP78/Bip and CHOP/GADD135 in a time-dependent manner. **(B)** DU-145 and PC-3 cells were pretreated with either NAC (20 μM), GSH (10 mM) or Catalase (20 nM) for 2 h and treated with Shikonin (2.5 μM) for 24 h and the indicated protein levels were measured using Western blotting. Representative data of three independent experiments are presented.

As the previous results suggested that Shikonin treatment induced intracellular Ca^2^,^+^ calpain activation and generation of ROS in prostate cancer cells (Figures 2 and 3), we therefore explored whether Shikonin can activate ROS dependent ER stress in prostate cancer cells. Recent studies have established that drug induced increase in ROS generation, intracellular Ca^2+^ and activation of ER stress leads to mitochondrial apoptosis in cancer cells [26-28]. Therefore, we further examined ER stress associated proteins in these cell lines. First, the phosphorylation patterns of PERK and eIF2α were assessed, since PERK, an ER-resident transmembrane kinase, is known to auto phosphorylate its cytoplasmic kinase domain in response to accumulated unfolded proteins in the ER lumen and activated PERK is subsequently capable of phosphorylating several cytosolic proteins including eIF2α [29-32]. Western blot analysis revealed that treatment of cells with Shikonin (2.5 μM) led to an increase in the phosphorylation of PERK up to 24 h and eIF2α for up to 9 h of treatment (Figure 4A). The expression of GRP78/Bip, which serves as a gatekeeper to the activation of ER stress transducers [33,34] was also examined. The results indicated that treatment with Shikonin significantly increased the expression of GRP78/Bip and CHOP/GADD135 and led to a decrease in the expression of procaspase-3 were ROS dependent manner (Figure 4B). These results collectively suggested that the intracellular ROS were directly involved in the regulation of ER stress induced by shikonin in prostate cancer cells.

### Shikonin regulates endoplasmic reticulum stress through modulation of intracellular calcium in prostate cancer cells

**Figure 5:**
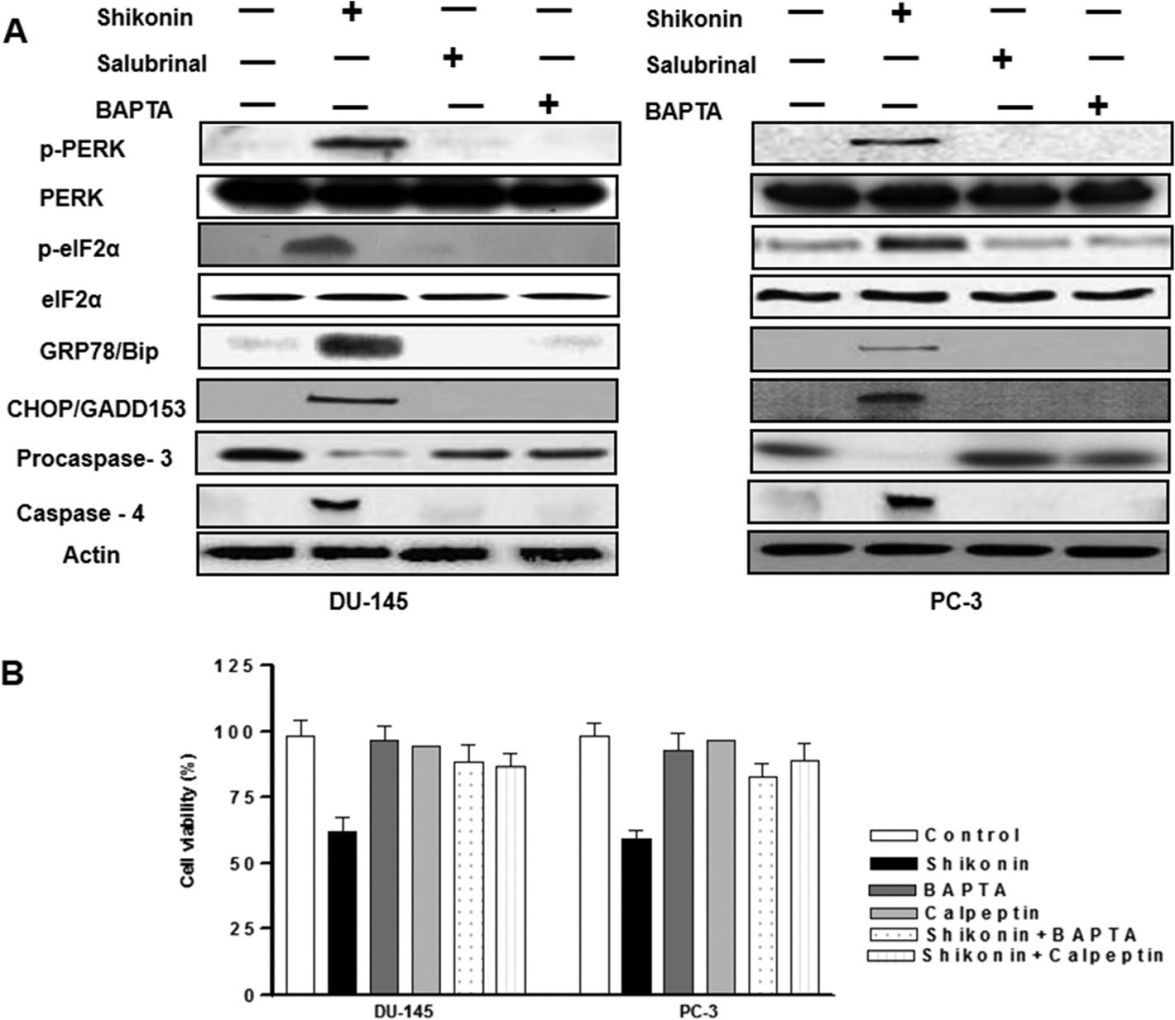
**Induction of ER stress is critical in Shikonin induced effects in DU-145 and PC-3 prostate cancer cells (A) Effect of Solubrinal or BAPTA on ER stress protein expression in DU-145 and PC-3 cells.** Protein expression of ER-stress was examined in pre-treated with BAPTA or Salubrinal in DU-145 and PC-3 cells, and western blot experiments were performed as described previously. **(B)** Inhibition of intracellular calcium or calpain reveses shikonin induced inhibition of cell viability. Cell viability was quantified using the CCK-8 assay in BAPTA or Calpeptin pretreated DU-145 and PC-3 cells. Data is expressed in means ± SEM and represents the results of three independent experiments.

We next assessed the expression of ER stress proteins as many studies have implicated the interplay between, ROS, mitochondria, intracellular Ca^2+^ and calpain activity determines ER stress and cell survival in cancer cells [32-34]. The results indicated that pretreatment with the Ca^2+^ chelator, BAPTA or the ER stress inhibitor Salubrinal inhibited the shikonin induced expression of p-pERK, p-pelF2α, CHOP/GADD153 and caspase 4 activity (Figure 5A). Furthermore, our cell proliferation data in presence of BAPTA (a Ca^2+^ chelator) or calpeptin (a calpain inhibitor) for 1h, also confirmed that Shikonin mediated cell death involves calcium signaling in prostate cancer DU-145 and PC-3 cells (Figure 5B).

### Inhibition of ER stress response by salubrinal attenuated shikonin induced effects

**Figure 6:**
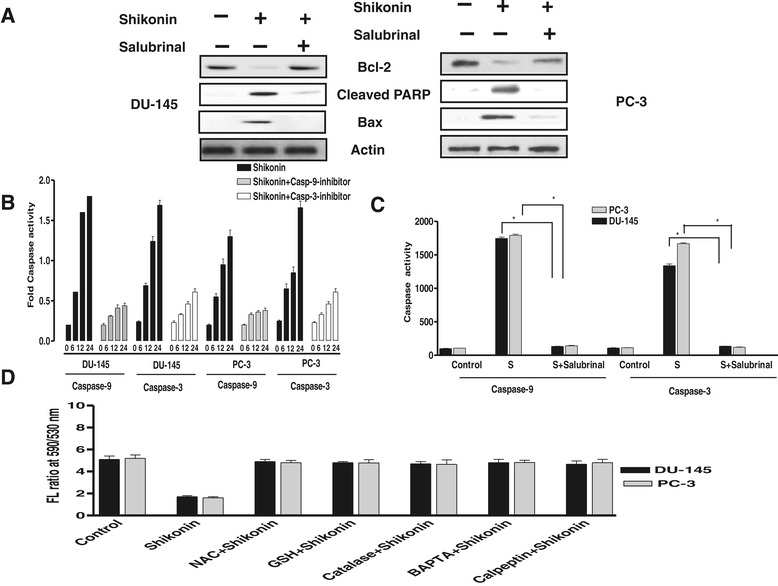
**ER stress inhibition attenuates Shikonin induced mitochondrial apoptosis. (A)** DU-145 and PC-3 cells were treated with either Shikonin (2.5 μM) alone for 24 h or pretreated with ER stress inhibitor Salubrinal (10 μM) for 24 h and subsequently treated with Shikonin. Protein expression was measured by western blotting as described in the Methods section. Shikonin treatment resulted in a marked reduction in the expression of Bcl-2 and an increase in the levels of Bax and cleaved PARP when compared with the controls. Pretreatment with ER stress inhibitor Salubrinal led to the increased expression of Bcl-2 and the decreased expression of Bax and cleaved PARP. **(B)** Pretreatment with caspase inhibitors reversed Shikonin induced caspase activation. Treatment of cells with Shikonin (2.5 μM) for 24 h resulted in a marked reduction of shikonin induced caspase-9 and caspase-3 activities. **(C)** Inhibition of ER suppressed shikonin induced casapase activity. Prostate cancer cells were pretreated with Salubrinal (10 μM), caspase 3 and 9 activity were performed by colorimetric assay as described in material and method section.** (D)** ER stress regulates mitochondrial membrane potential in Shikonin treated DU-145 and PC-3 cells. DU-145 and PC-3 cells were treated with either Shikonin (2.5 μΜ) alone or along with pretreatment with salubrinal (10 μM) for 24 h, and then incubated with mitochondria specific dye JC-1 (10 m g/ml aft 37°C for 15 min), and the florescence was monitored using fluorimeter (Excitation 530 nm/Fluorescence 590 nm) for measurement of the mitochondrial membrane potential. Shikonin treatment significantly reduced mitochondrial membrane potential whereas pretreatment of ER stress inhibitor reversed this effect. Data is expressed in means ± SEM and represents the results of three independent experiments.

**Figure 7:**
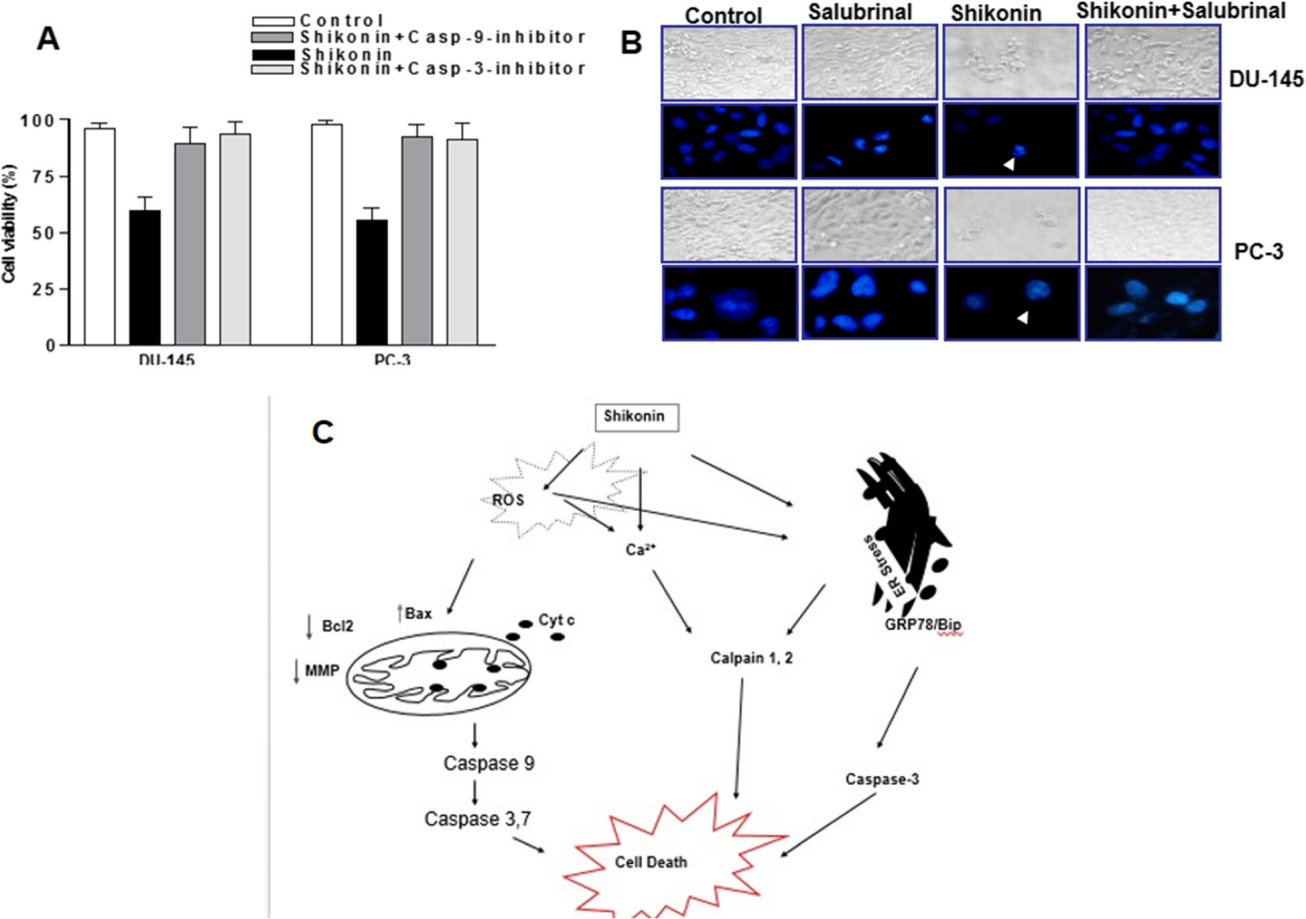
**Caspases and ER stress regulates Shikonin induced cell death in prostate cancer cells. (A)** Shikonin regulated caspase dependent cell death in DU-145 and PC-3 cells. Cells were preincubated with 50 μM of caspase-3 or 9 inhibitors, for 2 h, CCK-8 assay was performed after 48 h post treatment of Shikonin in these cells. Data is expressed in means ± SEM and represent the results of three independent experiments (p < 0.05). **(B)** Shikonin treatment induces apoptotic phenotype in prostate cancer cells. DU-145 and PC-3 cells were treated with 2.5 μM for 24 h, and their nuclear morphology was examined using DAPI staining in with or without salubrinal pre-incubated prostate cancer cells. In contrast to the control group, the group treated with Shikonin showed characteristic apoptotic phenotypes including heterogeneous staining, chromatin condensation, and DNA fragmentation. However the group pretreated with Salubrinal significantly reversed the Shikonin induced effect in both cell types. **(C)** The proposed shikonin induced cell death pathways in prostate cancer cells. Shikonin can directly influences intracellular calcium, and ER stress and activated ROS dependent mitochondrial apoptosis. Alternatively, Shikonin induced ROS may regulated intracellular calcium, and ER stress and cell death involving mitochondria and caspases in prostate cancer cells.

To investigate the mitochondrial apoptotic events involved in shikonin-induced apoptosis, the levels of the antiapoptotic protein Bcl-2 and the pro-apoptotic protein Bax were analyzed. Western blot analysis showed that the treatment of DU-145 and PC-3 cells with Shikonin resulted in a marked reduction in the expression of Bcl-2 and increased the level of Bax and cleaved PARP when compared with untreated cells (Figure 6A). These results suggested that shikonin alters the levels of pro- and anti-apoptotic proteins of the Bcl-2 family in a manner that contributes to the shikonin-induced apoptosis. Salubrinal is an inhibitor of the serine/threonine phosphatase PP1 and inhibits eIF2α dephosphorylation that in turn blocks ER stress-induced cell death [35,36]. The inhibition of ER stress in DU-145 and PC-3 cells by salubrinal led to the increased expression of Bcl-2 and decreased the levels of Bax and cleaved PARP, in contrast to cells treated with Shikonin alone, indicating that Shikonin treatment -induced the mitochondrial apoptotic pathway (Figure 6A). Similarly, pretreatment with caspase-9 and caspase-3 inhibitors (Figure 6B) or the ER stress inhibitor Salubrinal (Figure 6C) significantly inhibited the Shikonin induced caspase activation in DU-145 and PC-3 prostate cancer cells. Furthermore, cell viability assays with pretreatment of either the pan caspase inhibitor (Z-VAD-FMK) or the caspase-9 (Z-LEHD-FMK)or caspase-3 inhibitor (Z-DEVD-FMK) confirmed a sequential activation of caspases upon Shikonin treatment (Additional file 2: Figure S3). Further to define the role of ROS-Calcium Stress mediated modulation of mitochondrial membrane potential, we had pretreated prostate cancer with ROS scavengers (NAC,GSH or Catalase) and the calpain inhibitor calpeptin as described in material methods. Our results suggested that both ROS and intracellular calcium signaling are crucial for mitochondrial apoptosis in prostate cancer cells. Additionally we also observed that either inhibition of caspase 9, 3 or Salubrinal prevented Shikonin induced reduction of cell viability (Figure 7A), and formation of apoptotic bodies in these cells (Figure 7B) as a marker of apoptotic cell death. Collectively these results indicated that ER stress played an critical role in the shikonin induced apoptosis of prostate cancer cells.

## Discussion

Shikonin effectively induces apoptosis, necrosis and necroptosis in cancer cells [10,14,23]. Shikonin is known to selective killing of prostate cancer cell types, while sparing normal cells [14]. However nothing was known regarding the effects of Shikonin on regulation of the ROS-Ca^2+^-pro apoptotic-ER stress in prostate cancer cells. Using *in vitro* assays we report that the natural naphthoquinone Shikonin induces cell death in DU-145 and PC-3 prostate cancer cell lines through the modulation of the ER stress and the mitochondrial apoptotic pathway. Furthermore, for the first time showed that Shikonin induces apoptosis in prostate cancer cells through the ROS mediated-intracellular Ca^2+^-proapoptotic ER stress associated mitochondrial dysfunction.

Previous studies have established that increase in the intracellular Ca^2+^levels lead to the activation of ROS and is considered as a second messenger in ER stress signaling [37]. In this study, treatment of DU-145 and PC-3 cells with Shikonin resulted in marked increase in the levels of intracellular Ca^2+^ within 60 min and ROS within 90 min. The majority of Ca^2+^ remains in the ER, but ER stress leads to the release of Ca^2+^ [37]. Moreover, pretreatment of cells with antioxidants like NAC, GSH or Catalase markedly decreased the levels of intracellular Ca^2+^, suggesting that intracellular ROS were directly involved in the cytotoxic action of Shikonin (Figures 2 and 3). These data indicated that Shikonin induces production of ROS and activates ER stress and the subsequent release of Ca^2+^ in both DU-145 and PC-3 cells lead to cell death. Our result is consistent with previous reports that ROS activates ER dependent apoptosis in cancer cells [27-29]. Our results also indicated that the treatment of these cells with Shikonin induceds ER stress leading to the elevation in the levels of GRP78/Bip and CHOP/GADD153, and the phosphorylation of PERK and eIF2α (Figure 4A), while pretreatment with antioxidants attenuated these effects (Figure 4B).

The members of the Bcl-2 family of proteins are important regulators of apoptotic cell death [38]. Although the involvement of Bcl-2 proteins in ER stress-induced cell death is clear, the mechanism by which they are regulated by ER stress is not well understood. Until recently, Bcl-2 proteins were thought to regulate the mitochondrial-mediated apoptotic pathway exclusively [38,39]. One of the recent studies linked ER stress-induced cell death to the Bcl-2 family of proteins showed that overexpression of Bcl-2 or the deficiency of Bax and Bak conferred protection against lethal ER stress [38]. Stress signals are relayed from the ER to mitochondria, and ER stress induced apoptosis, similar to mitochondrial-mediated apoptosis regulated by the Bcl-2 family of proteins [39]. The ratio of the Bax/Bcl-2 is critical for the induction of apoptosis [40]. ER stress inducers including cellular stress inducers have also been shown to induce a change in the conformation of Bax, resulting in its accumulation on the mitochondria, and to induce the release of cytochrome c from the mitochondria into the cytosol [31,41,42]. Cytosolic cytochrome c then binds to Apaf-1, leading to the activation of caspase-3 and PARP protein [43]. The present results also indicate that the reduced expression of antiapoptotic Bcl-2 protein and the increased expression of the pro-apoptotic Bax protein facilitated the Shikonin-mediated cell death of these cells (Figure 6), which increases the ratio of Bax/Bcl-2. This may be responsible for the concomitant execution phase of apoptosis observed in these cells, which included the disruption of the mitochondrial membrane (Figure 6). As the level of cytochrome c increases in the cytosol, it leads to the activation of the procaspase-9 and caspase- 3 [44]. Activated caspase-3 is the key executioner of apoptosis and the cleaved caspase-3 leads to the cleavage and inactivation of key cellular proteins such as PARP [45,46]. The present results revealed that the treatment of prostate cancer cells with Shikonin led to the activation of caspase-9, caspase-3, and PARP (Figure 1). Salubrinal was identified as a selective inhibitor of phosphatases that act on eIF2α thereby maintaining protein phosphorylation and offering protection from the adverse effects of ER stress. Inhibition of ER stress in DU-145 and PC-3 cells by salubrinal in the present study led to the increased expression of Bcl-2 and caspase-9, and caspase-3 activities and the decreased expression of Bax (Figure 6B), caspase dependent cell death (Figure 7A) and DNA damage as evident by the nuclear condensation indicated by DAPI staining (Figure 7B). Natural compounds like shikonin might have multiple cellular targets in order to achieve their biological beneficial effects such as tumor growth inhibition [14,16]. A previous study using prostate cancer cells had indicated that the tumor proteasomal chymotrypsin subunit is one of the cellular as well as *in vivo* biological targets of shikonin [46]. Our results are consistent with is study as it demonstrates that shikonin with established proteasomal inhibitory activity induces apoptosis through induction of reactive oxygen species and endoplasmic reticulum stress-in prostate cancer cells [47] (Figure 7C).

## Conclusion

Taken together our studies suggest that (i) Shikonin inhibits proliferation of hormone refractory prostate cancer cells through the induction of ROS, mediated activation of ER stress and intracellular Ca^2+^, and (ii) induction of mitochondrial apoptotic pathway mediated through the enhanced expression of Bax, disruption of the mitochondrial membrane potential, PARP cleavage and activation of caspase-9 and caspase-3. This ability of Shikonin to induce dual pathways of cell death underscores its potential as a chemotherapeutic agent against hormone refractory prostate cancers need for further evaluation using *in vivo* models.

## Additional files

Additional file 1: Table S1. Summary of cell lines used in this study. Additional file 2: Figure S1. Shikonin treatment inhibits cell viabilities of multiple cancer cells while sparing normal cell types. (A) Transformed cells of diverse origin (Ishikawa: endometrial adenocarcinoma, C33A: cervical cancer cells, LN229: human glioblastoma cancer cells and CAL 27: oral adenosquamous cells) were treated with various doses of shikonin (0.5,1, 2.5, 5 and 10 μM) for 24 h and the cell viabilities were assayed using CCK-8 assay. (B) Nontransformed cells of diverse origin, i.e. oral keratinocytes; human astrocytes; human mammary epithelial cells (HMEC); and peripheral blood mononuclear cells (PBMCs) were treated with various doses of shikonin (0.5,1, 2.5, 5 and 10 μM) for 24 h and cell viabilities were assayed using CCK-8 assay. Data is expressed in means ± SEM and represent the results of three independent experiments (*p < 0.05). **Figure S2.** Shikonin treatment inhibits cellular proliferation of prostate cancer cells while sparing normal prostate epithelial cells (PrECs). Normal prostate epithelial (PrEC) or prostate cancer cells (DU-145 or PC-3) were treated with various doses of shikonin (0.5,1, 2.5, 5 and 10 μM) for 24 h and the cell proliferation was quantified through bromodeoxyuridine (BrdU) incorporation into DNA using a nonradioactive colorimetric assay as described in the materials and methods section. Data is expressed in means ± SEM and represent the results of three independent experiments (*p < 0.05). **Figure S3.** Inhibition of caspase activity (Pan caspase, caspase-9 and caspase-3 activities) reverses Shikonin induced inhibition of cell viability in prostate cancer cells, indicating sequential activation of caspases. Prostate cancer cells (DU-145 or PC-3) were preincubated with caspase inhibitors (Pan caspase Inhibitor: Z-VAD-FMK, Caspase −9 Inhibitor: Z-LEHD-FMK and Caspase −3 Inhibitor: Z-DEVD-FMK at a dose of 20 μM) alone or in combination for 2 h and subsequently treated with Shikonin for 24 h. Subsequently, cell viabilities were assayed using CCK-8 assay. Data is expressed in means ± SEM and represent the results of three independent experiments (*p < 0.05).
