# Supplementary material for: Shikonin selectively induces apoptosis in human prostate cancer cells through the endoplasmic reticulum stress and mitochondrial apoptotic pathway
Source: J Biomed Sci. 2015 Apr 1;22(1):26. doi: 10.1186/s12929-015-0127-1 (PMC4389804; doi:10.1186/s12929-015-0127-1)
Supplement: Additional file 1: Table S1. — Summary of cell lines used in this study. [file 12929_2015_127_MOESM1_ESM.doc]

**Additional file 1: Table S1**

Summary of cell lines used in this study

| **Cell Lines** | **Source of cell lines** | **Origin** |
| --- | --- | --- |
| DU-145 | ATCC, Cat. No: HTB-81 | Human Prostate, adenocarcinoma |
| PC-3 | ATCC, Cat. No: CRL-1435 | Human Prostate, adenocarcinoma |
|  | ScienCell Research Laboratories  Cat. No: 1800 | human brain (cerebral cortex) |
| Human Oral Keratinocytes (HOK) | ScienCell Research Laboratories,  Cat. No: 2610 | human oral mucosa |
| HMEC | Lonza; Cat. No: CC-2551 | Human Mammary Epithelial Cells |
