# Supplementary material for: Shikonin selectively induces apoptosis in human prostate cancer cells through the endoplasmic reticulum stress and mitochondrial apoptotic pathway
Source: J Biomed Sci. 2015 Apr 1;22(1):26. doi: 10.1186/s12929-015-0127-1 (PMC4389804; doi:10.1186/s12929-015-0127-1)

Supplementary Figure S1

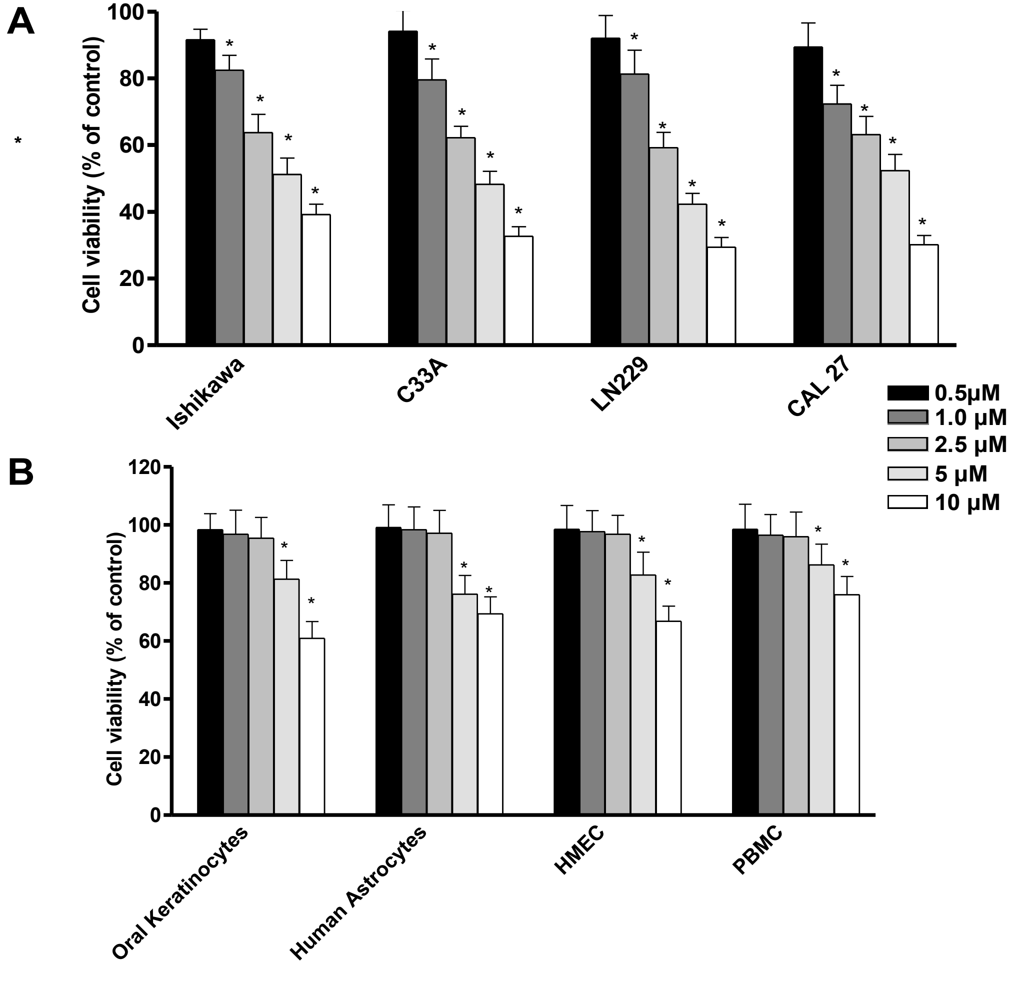

Supplementary Figure S2

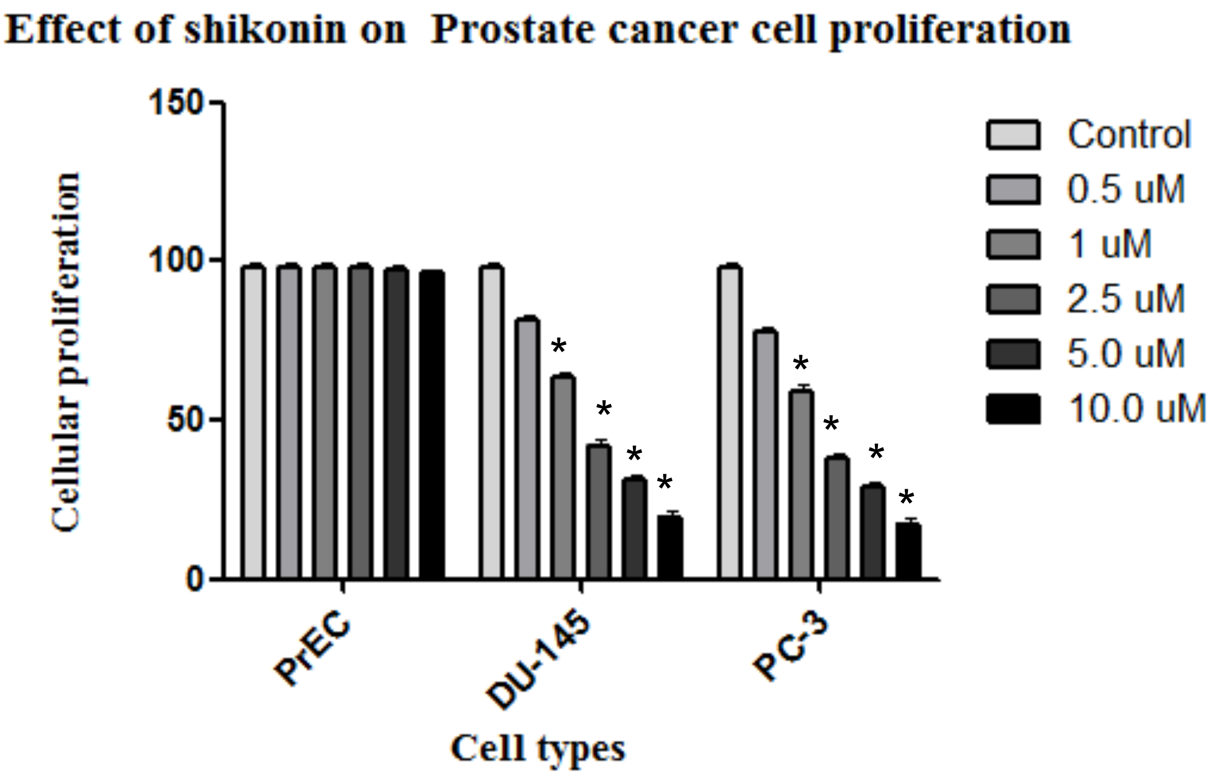

Supplementary Figure S3

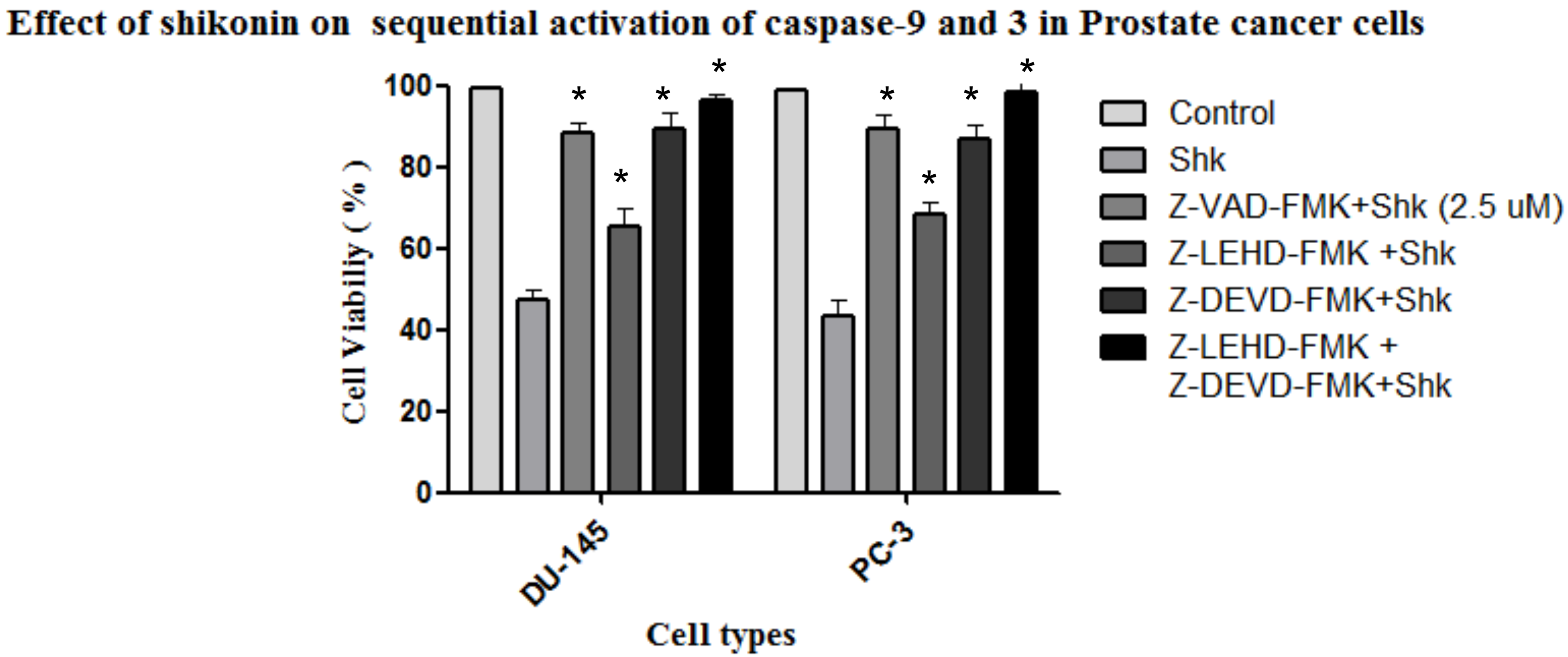

Supplement: Additional file 2: Figure S1. — Shikonin treatment inhibits cell viabilities of multiple cancer cells while sparing normal cell types. (A) Transformed cells of diverse origin (Ishikawa: endometrial adenocarcinoma, C33A: cervical cancer cells, LN229: human glioblastoma cancer cells and CAL 27: oral adenosquamous cells) were treated with various doses of shikonin (0.5,1, 2.5, 5 and 10 μM) for 24 h and the cell viabilities were assayed using CCK-8 assay. (B) Nontransformed cells of diverse origin, i.e. oral keratinocytes; human astrocytes; human mammary epithelial cells (HMEC); and peripheral blood mononuclear cells (PBMCs) were treated with various doses of shikonin (0.5,1, 2.5, 5 and 10 μM) for 24 h and cell viabilities were assayed using CCK-8 assay. Data is expressed in means ± SEM and represent the results of three independent experiments (*p < 0.05). Figure S2. Shikonin treatment inhibits cellular proliferation of prostate cancer cells while sparing normal prostate epithelial cells (PrECs). Normal prostate epithelial (PrEC) or prostate cancer cells (DU-145 or PC-3) were treated with various doses of shikonin (0.5,1, 2.5, 5 and 10 μM) for 24 h and the cell proliferation was quantified through bromodeoxyuridine (BrdU) incorporation into DNA using a nonradioactive colorimetric assay as described in the materials and methods section. Data is expressed in means ± SEM and represent the results of three independent experiments (*p < 0.05). Figure S3. Inhibition of caspase activity (Pan caspase, caspase-9 and caspase-3 activities) reverses Shikonin induced inhibition of cell viability in prostate cancer cells, indicating sequential activation of caspases. Prostate cancer cells (DU-145 or PC-3) were preincubated with caspase inhibitors (Pan caspase Inhibitor: Z-VAD-FMK, Caspase −9 Inhibitor: Z-LEHD-FMK and Caspase −3 Inhibitor: Z-DEVD-FMK at a dose of 20 μM) alone or in combination for 2 h and subsequently treated with Shikonin for 24 h. Subsequently, cell viabilities were assayed using CCK-8 assay. Dat [file 12929_2015_127_MOESM2_ESM.pdf]
